# Classification and Detection of COVID-19 and Other Chest-Related Diseases Using Transfer Learning

**DOI:** 10.3390/s22207977

**Published:** 2022-10-19

**Authors:** Muhammad Tahir Naseem, Tajmal Hussain, Chan-Su Lee, Muhammad Adnan Khan

**Affiliations:** 1 Department of Electronic Engineering, Yeungnam University, Gyeongsan 38541, Korea; 2 Riphah School of Computing & Applied Sciences (RSCI), Riphah International University, Lahore 55150, Pakistan

**Keywords:** COVID-19, pneumothorax, pneumonia, tuberculosis, deep learning, convolutional neural networks, transfer learning

## Abstract

COVID-19 has infected millions of people worldwide over the past few years. The main technique used for COVID-19 detection is reverse transcription, which is expensive, sensitive, and requires medical expertise. X-ray imaging is an alternative and more accessible technique. This study aimed to improve detection accuracy to create a computer-aided diagnostic tool. Combining other artificial intelligence applications techniques with radiological imaging can help detect different diseases. This study proposes a technique for the automatic detection of COVID-19 and other chest-related diseases using digital chest X-ray images of suspected patients by applying transfer learning (TL) algorithms. For this purpose, two balanced datasets, Dataset-1 and Dataset-2, were created by combining four public databases and collecting images from recently published articles. Dataset-1 consisted of 6000 chest X-ray images with 1500 for each class. Dataset-2 consisted of 7200 images with 1200 for each class. To train and test the model, TL with nine pretrained convolutional neural networks (CNNs) was used with augmentation as a preprocessing method. The network was trained to classify using five classifiers: two-class classifier (normal and COVID-19); three-class classifier (normal, COVID-19, and viral pneumonia), four-class classifier (normal, viral pneumonia, COVID-19, and tuberculosis (Tb)), five-class classifier (normal, bacterial pneumonia, COVID-19, Tb, and pneumothorax), and six-class classifier (normal, bacterial pneumonia, COVID-19, viral pneumonia, Tb, and pneumothorax). For two, three, four, five, and six classes, our model achieved a maximum accuracy of 99.83, 98.11, 97.00, 94.66, and 87.29%, respectively.

## 1. Introduction

In December 2019, a new virus caused by SARS-CoV-2 was identified in Wuhan, China. This coronavirus (COVID-19) elicits severe acute and Middle East respiratory syndromes. Coronaviruses belong to a large family of viruses that cause respiratory infections and are extremely contagious. Owing to rapid spread of SARS-CoV-2 worldwide, the World Health Organization (WHO) declared a pandemic [1,2]. Many people have died because of this virus, and it has disrupted many countries’ economies. Symptoms of this disease include respiratory problems, fever, cough, and pneumonia. Diagnosing this virus quickly is difficult because its symptoms are similar to those of other pulmonary diseases, such as viral pneumonia, bacterial pneumonia, and tuberculosis (Tb). To diagnose COVID-19, experts perform a testing technique called the reverse transcription polymerase chain reaction, which is expensive and time consuming [3]. Computed tomography (CT) and chest radiography are the most important methods for disease diagnosis. Chest radiography is an easy and quicks means for diagnosing coronaviruses [3]. Currently, detecting chest-related diseases using X-ray images is challenging because experienced radiologists are required. The manual detection of diseases by radiologists is difficult, and its error rate is therefore high [4]. Furthermore, training a radiologist is time consuming and expensive. In addition, a delayed diagnosis can be harmful to patients [5]. The development of a computer-aided detection system is urgent because it enables the early detection of diseases, saves money and time, and decreases the chance of death for an infected person. Privacy and security are major concerns for healthcare systems owing to electronic health records. Researchers have focused on blockchain technology to improve the security of healthcare systems [6].

Convolutional neural networks (CNNs) have been applied in the medical science field [7,8,9] to solve different problems related to chest X-ray images and CT scans [10,11,12,13,14,15,16]. The main issue with CNNs is that their use requires a large dataset and considerable time with GPU support for training. Transfer learning (TL) is frequently used to address the need for large datasets. Several pretrained CNN models have been used for COVID-19 detection, including DenseNet201 [4], ResNet50 [14], ResNet101 [4], VGG16 [17,18], VGG19 [18,19], Chex-Net [4], DarkCovidNet [20], MobileNet-V2 [4], and NasNet-Mobile [18]. To train a pretrained model, the ImageNet [21] dataset has been used, which contains over a million images with 1000 classes.

Chowdhury et al. evaluated eight pretrained models: MobileNet_v2, a CNN model that was 53 layers deep, SqueezeNet with 18 layers, ResNet18 with 18 layers, Inception_v3 with 48 layers, ResNet101 with 101 layers deep, ChexNet, with 121 layers, VGG19, with 19 layers, and DenseNet201 with 201 layers. The dataset contained 423 images of COVID-19, 1485 images of viral pneumonia, and 1579 normal X-ray images. The scheme exhibited a detection accuracy of 99.7% for the two-class problem and 99.55% for the three-class problem [4]. Asif et al. evaluated Inception_v3 using a pretrained model to detect COVID-19 and pneumonia. They used a dataset containing 864 images of COVID-19, 1345 images of viral pneumonia, and 1341 images of normal X-rays. The detection accuracy achieved by their model exceeded 98% [22]. Ozturk et al. proposed DarkCovidNet, which typically consists of five pooling layers and 19 conversion layers for binary and multi-classification. The binary classes were COVID-19 and “no findings,” and the multiple classes were COVID-19, “no findings,” and pneumonia. The dataset consisted of 127 images of COVID-19, 500 images of pneumonia, and 500 images with no findings. A detection accuracy of 87.02% was achieved using five-fold cross validation [20]. Panwar et al. discussed a model for predicting COVID-19 using chest X-ray images with nCovnet, which is an alternate fast method for COVID-19 detection, for a two-class dataset of 142 images of COVID-19 and 142 images of healthy subjects. The detection accuracy of this model was 88% [23]. CoroNet, a model based on the Xception CNN architecture, 71 layers deep, was proposed to predict and detect COVID-19 using X-ray images of a four-class dataset containing 330 images of bacterial pneumonia, 310 normal images, 327 images of viral pneumonia, and 284 COVID-19 images. The dataset consisted of four balanced classes. An accuracy of 89.6% was achieved with four-fold cross-validation using the Google Collaborator Ubuntu server and a Tesla K80 graphics card [24]. Das et al. used the best version of the inception (xception) model. The dataset consisted of 127 images of COVID-19, 500 images of pneumonia, and 500 images of COVID-19 negative and other infectious diseases. They used 70% of the data for training, and the remaining 30% were divided into 10% for validation and 20% for testing. The accuracy of the model was 98.64% [25].

Wang et al. [26] discussed the first model designed for COVID-19 detection (COVID-net), which generated 13,975 CXR images from 13,870 patients. Ismael et al. [27] proposed a deep-learning approach for binary classification to detect COVID-19 by using pretrained models like ResNet18, ResNet50, ResNet101, VGG16, and VGG19. Support Vector Machines (SVM) classifier with various kernel functions, namely Linear, Quadratic, Cubic, and Gaussian was used to classify deep features. The dataset used 180 images for COVID-19 and 200 images for normal. ResNet50 achieved an accuracy of 94.7%. 

Vaid et al. used a modified version of VGG19 to detect COVID-19 from chest X-ray images by splitting the dataset: 80% for training, 10% for validation, and 10% for testing. The model used a publicly available dataset from different countries. The accuracy achieved by the model was 96.3% [28].

Farooq et al. presented a COVID-ResNet model that uses a pretrained ResNet50 architecture fine-tuned to improve the performance of the model and minimize the training time. The model performed multi-class classification for three infection types with normal individuals and achieved an accuracy of 96.23% [29].

Masko et al. used the CIFAR-10 dataset containing 60,000 images of 10 classes to create training sets with different distributions between classes. Some sets contained many images of one class, whereas other classes contained few images. These sets were used to train a CNN, and the classification performance was then measured for each training set. The results show that unbalanced training data can have a severely negative impact on overall performance, whereas the balanced training data yielded the best results [30]. Data unbalancing presents classification problems because algorithms trained with balanced datasets outperform those trained with unbalanced datasets. Several studies have empirically demonstrated that balanced datasets outperform unbalanced datasets [31,32].

Chan et al. used an SVM to detect pneumothorax, which is a chest-related disease. The model used local binary pattern (LBP) to extract features and, after extracting the features, the SVM was used to detect pneumothorax. This model achieved an accuracy of 82.2% [33]. 

Extensive, previous studies were conducted with only a limited amount of COVID-19 data and by classifying only two or three types of chest-related diseases with unbalanced datasets, including viral pneumonia, COVID-19, bacterial pneumonia, and normal cases. Unbalanced datasets do not achieve optimal results for unbalanced classes. Our study investigated different chest-related diseases, including COVID-19, bacterial pneumonia, Tb, viral pneumonia, and pneumothorax, by creating and using balanced datasets and dividing the problem into five classes, thereby requiring two-class, three-class, four-class, five-class, and six-class classifiers. The proposed scheme was compared with existing state-of-the-art schemes, and performed well in terms of accuracy.

### Limitations of Related Work and Our Contributions

Table 1 lists the problems with existing techniques. These techniques have at least one of the following shortcomings:

- Despite good accuracy, the models presented in [4,14,26] used unbalanced classes.
- The models in [20,23,24,27] were tested with unbalanced classes and yielded low accuracy.

Unlike previous studies, our proposed approach does not rely on unbalanced datasets but uses balanced datasets. In addition, the proposed model presents multiclass classification for five diseases with good accuracy. The major contributions of this study are as follows:

- Two-class and multi-class classification with different combinations of chest-related diseases, including COVID-19, normal, viral pneumonia, bacterial pneumonia, tuberculosis, and pneumothorax, are proposed using different pretrained CNN-based models.
- Two balanced datasets were used to classify five chest-related diseases.
- Different image augmentation techniques were used to balance or increase the dataset size.
- The proposed model was compared with state-of-the-art methods and performed well.

The remainder of this paper is organized as follows. Section 2 describes the materials and methods used in this study. Section 3 describes the experiment setup and performance measures used. Section 4 presents results and discussions, and Section 5 concludes the paper and discusses future work.

## 2. Materials and Methods

This section first discusses the procedure for constructing datasets to detect COVID-19 and other chest-related diseases, and then the data augmentation techniques used. Subsequently, TL using CNNs is presented, and the proposed model is discussed.

### 2.1. Datasets

In this study, two datasets, Dataset-1 and Dataset-2, were constructed from four sources. Dataset-1 was a balanced dataset for three diseases and the normal case, which was obtained from three different sources, as shown in Table 2. Dataset-2 was a balanced dataset for five diseases and the normal case, which was obtained from four different sources, as shown in Table 2.

The first source (https://www.kaggle.com/datasets/tawsifurrahman/covid19-radiography-database, accessed on 25 July 2022) provided 3616 COVID-19 images and 10,192 normal images, as discussed in [4]. We randomly selected 1500 images for the COVID-19 and normal cases for Dataset-1 and 1200 images for the COVID-19 and normal cases for Dataset-2. Viral pneumonia, bacterial pneumonia, and normal case images were obtained from a second dataset (https://www.kaggle.com/datasets/paultimothymooney/chest-xray-pneumonia, accessed on 25 July 2022) of 5863 images, as discussed in [34]; 1352 viral pneumonia images were available. We applied image augmentation to balance the dataset and generated 148 additional images for Dataset-1. To construct Dataset-2, we randomly selected 1200 images for each class of viral pneumonia and bacterial pneumonia.

The third source (https://www.kaggle.com/datasets/kmader/pulmonary-chest-xray-abnormalities, accessed on 25 July 2022) of images was Tb, which was a dataset of 394 images. Tb dataset images were collected from two sources: 336 images from China and 58 images from Montgomery County, MD, USA [35,36]. To balance Dataset-1, image augmentation was applied to randomly selected images; thus, we obtained 1106 more images and a total of 1500 images for Dataset-1. To construct Dataset-2, we randomly selected 1200 images from the Tb case from Dataset-1. This study used balanced datasets because these would allow the model to generate a higher accuracy and balanced detection rate. However, an unbalanced dataset may influence performance and error rate, which may fluctuate or increase when splitting unbalanced data. Some batches may contain only negative instances because of the skewed distribution of the training data; thus, the trained models perform poorly when applied to test data [37,38].

**Figure 1:**
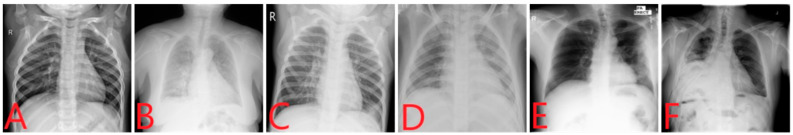
Sample images of different diseases: (**A**) Normal, (**B**) COVID-19, (**C**) Viral pneumonia, (**D**) Bacterial pneumonia, (**E**) Tb, and (**F**) Pneumothorax.

The fourth and last source (https://www.kaggle.com/c/siim-acr-pneumothorax-segmentation, accessed on 25 July 2022) of images was Pneumothorax, which is a collection of 12,047 images, as discussed in [39]. We randomly selected 1200 images of pneumothorax for Dataset-2. For the two-class, three-class, and four-class datasets, we used Dataset-1, and for the six-class dataset, we used Dataset-2. After compiling all images, a balanced four-class dataset was constructed, i.e., Dataset-1, which contained 6000 images and 1500 images for each class including COVID-19, viral pneumonia, Tb, and normal. Then, another balanced dataset was constructed, Dataset-2, which contained 7200 images and 1200 images for each class, including COVID-19, viral pneumonia, bacterial pneumonia, tuberculosis, pneumothorax, and normal. Both datasets contained more images than those used in previous studies [4,20,24,40]. Figure 1 shows samples of X-ray images used in this study. These images are (A) Normal, (B) COVID-19, (C) Viral pneumonia (D) Bacterial pneumonia (E) Tb, and (F) Pneumothorax.

### 2.2. Data Augmentation

The main problem with image datasets is overfitting. Augmentation was applied to reduce overfitting and artificially enlarge the dataset [41]. Data augmentation is a technique used to increase the volume of data, thereby improving the generalization of the training model and reducing overfitting. In this study, we performed different augmentation techniques, such as 5° image rotations, range shearing, range zooming, and horizontal flips.

Dataset-1 had four classes: normal, COVID-19, viral pneumonia, and Tb, but the Tb and viral pneumonia classes had fewer images than the others. For viral pneumonia, only 1352 unaugmented images were available; therefore, we applied augmentation and generated 148 additional images. For Tb, 394 images were available; therefore, we applied augmentation and generated 1106 additional images.

Dataset-2 had six classes: normal, COVID-19, viral pneumonia, bacterial pneumonia, Tb, and pneumothorax, but Tb had fewer images than the other classes. We randomly selected 1200 Tb images from Dataset-1. Because of the limited computational resources required to balance the dataset, we selected 1200 unaugmented images from the original sources for the other classes.

### 2.3. Transfer Learning (TL) Using CNNs

CNNs have been used to recognize patterns, detect objects, recognize faces using images, as well as recognize diseases in medical images. CNNs and fully connected networks are fundamental components of deep learning. The main objective of a CNN is to learn features from images and data [42]. A CNN is trained for a specific task using a large dataset, and the CNN then learns and extracts important features from images. In the next phase, a pretrained CNN is applied to a new set of images, features are extracted based on previous knowledge, and all extracted features are used in a new network to perform a classification task [19]. This method reduces the training cost because training from scratch is time-consuming and requires powerful computational resources.

TL is used to solve different but related tasks using new data. TL can be used as an application of CNNs, in which the dataset is limited or small. TL has been used to detect COVID-19 and other chest-related diseases in chest X-ray images [12,13,14,19,20,24,26]. TL techniques use the large ImageNet [21] dataset to train the model for applications with a small dataset. In addition, TL is helpful in medical applications for which collecting a large dataset is difficult.

### 2.4. Proposed Model

**Figure 2:**
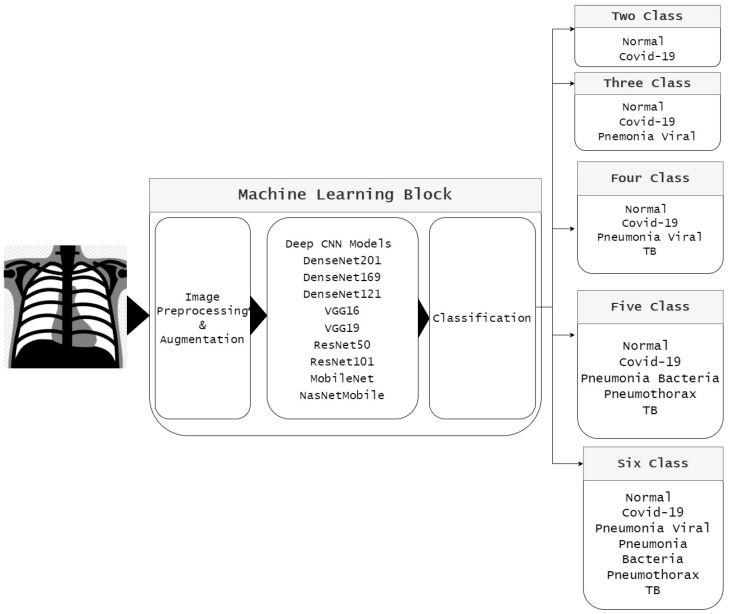
Pipeline of proposed model.

Figure 2 shows a block diagram of the proposed model used to classify COVID-19 and other chest-related diseases. The classification problem is divided into five classes: two-class classifier (normal and COVID-19), three-class classifier (normal, COVID-19, and viral pneumonia), four-class classifier (normal, COVID-19, viral pneumonia, and Tb), five-class classifier (normal, COVID-19, Tb, bacterial pneumonia, and pneumothorax), and six-class classifier (normal, COVID-19, viral pneumonia, Tb, bacterial pneumonia, and pneumothorax). The proposed model is divided into three steps: dataset preprocessing, use of listed pretrained networks, and final selection of pretrained models.

First, image augmentation was applied to increase the size of the dataset for classes with fewer images, which helps create balanced datasets [43]. Second, different transformations, such as 5° image rotations, range shearing, range zooming, and horizontal flips, were used to increase the size of the dataset. During preprocessing, all images were resized to 224 × 224 × 3 pixels to match the requirements of all networks.

Second, nine different pretrained state-of-the-art CNN models were used, ResNet50 [14,18], ResNet101 [4,18], VGG16 [17,18], VGG19 [18,19], MobileNet _v2 [4,18], NasNet-mobile [18], DenseNet201 [4,18], DenseNet169 [44], and DenseNet121 [45], which were already trained using ImageNet [21], and accuracy and other performance measures were calculated for all models. Finally, the best pretrained model was selected from the individual classes, which was then used to validate the datasets.

## 3. Experiment Setup and Performance Measures

All experiments were performed using the online free resource Google Collaborator (Colab) with a Tesla K80 and T4 GPU to overcome computational issues. The CNN models were pretrained with ImageNet [21] weights using a categorical cross-entropy function. The dropout, batch size, and number of epochs were set to 0.5, 32, and 50, respectively, for all experiments. The dataset was randomly divided into 80% for training and 20% for testing. For every pretrained model, we trained only the last layer. The input shape was 224 × 224 × 3. Pooling was set to max-pooling. The model was initially trained on ImageNet weights, and the activation function used was softmax. To measure the performance of the proposed model, accuracy, specificity, sensitivity precision, and *F*2 *score* were used as performance measures, which are given as follows:

(1) $$Accuracy=\frac{TP+TN}{TP+TN+FP+FN}$$

(2) $$Specificity=\frac{TN}{TN+FP}$$

(3) $$Sensitivity\left(Recall\right)=\frac{TP}{TP+FN}$$

(4) $$Precision=\frac{TP}{TP+FP\;}$$

(5) $$F2\;Score=\frac{5*Precision*Recall}{4*Precison+Recall}$$

## 4. Results and Discussion

This study evaluated nine different TL models (Densenet201, Densenet169, Densenet121, VGG16, VGG19, ResNet50, ResNet101, NasNet-Mobile, and MobileNet_v2) to detect COVID-19 and other chest-related diseases. Different combinations of classes were considered to classify different diseases. Table 3 lists the details of the classifiers for different disease types.

### 4.1. Two-Class Classifier

**Figure 3:**
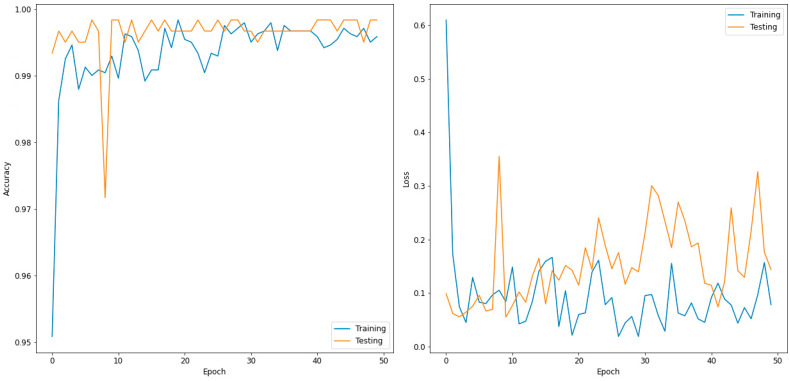
Accuracy (**left**) and loss curve (**right**) of DenseNet169 for two-class classifier.

Table 4 presents the evaluated confusion matrix for the two-class classifier (normal and COVID-19) and DenseNet169, which outperformed the other models. In total, 600 images were used in the validation phase. These were further divided into 300 input images each for the normal and COVID-19 disease types. For the normal case, 300 images were correctly predicted as normal, and no image was predicted as COVID-19. Similarly, 299 images were predicted correctly for COVID-19, and one image was incorrectly predicted as normal. Table 5 shows the performance metrics for the best-performing model for the two classes (DenseNet169). Figure 3 shows the accuracy and loss curves of the DensNet169 model for a two-class classifier for 50 epochs.

### 4.2. Three-Class Classifier

**Figure 4:**
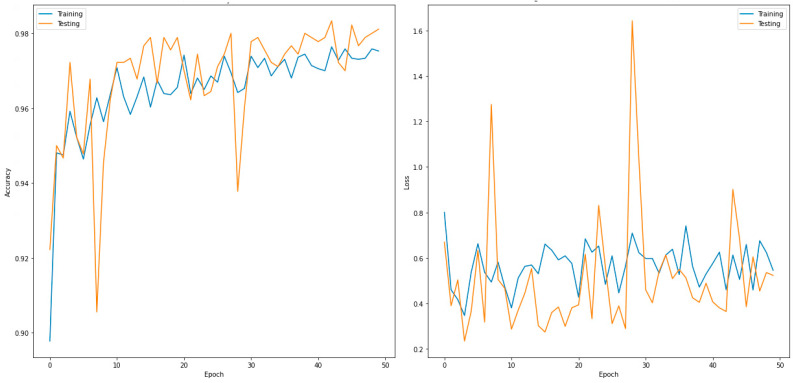
Accuracy (**left**) and loss curves (**right**) of DenseNet201 for three-class classifier.

This section discusses the evaluated confusion matrix for the three-class classifier (normal, COVID-19 and viral pneumonia). Table 6 shows the validation confusion matrix for DenseNet201, which outperformed the other models. In total, 900 images were used for the validation phase. These were further divided into 300 input images for the normal, COVID-19, and viral pneumonia cases each. For the normal case, 291 images were correctly predicted as normal, no image was predicted as COVID-19, and nine images were predicted as viral pneumonia. For COVID-19, 297 images predicted correctly, one was incorrectly predicted as normal, and two were misclassified as viral pneumonia. Similarly, for viral pneumonia, 295 images predicted correctly, only five images were misclassified as normal, and no image was misclassified as COVID-19. Table 7 shows the performance metrics for the best-performing model for the two classes (DenseNet201). Figure 4 shows the accuracy and loss curves of the DensNet201 model for the two-class classifier for 50 epochs.

### 4.3. Four-Class Classifier

**Figure 5:**
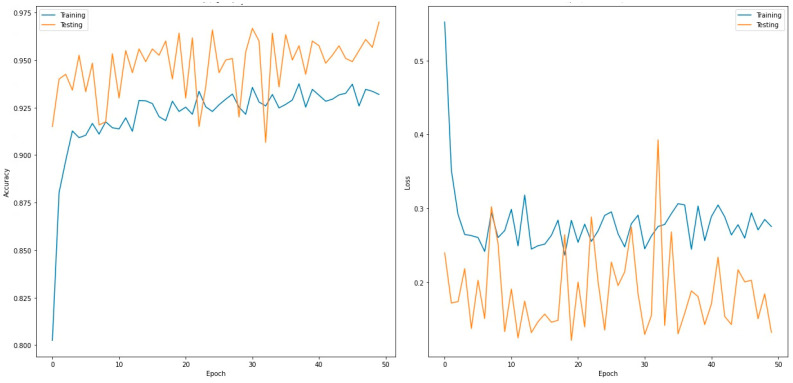
Accuracy (**left**) and loss curves (**right**) of VGG19 for four-class classifier.

This section presents the evaluated performance of the four-class classifier (normal, COVID-19, viral pneumonia, and TB). Table 8 presents the validation confusion matrix for the VGG19 model, which outperformed the other models. In total, 1200 images were used during the validation phase. These were further divided into 300 input images each for normal, COVID-19, viral pneumonia, and TB. For the normal case, 291 images were correctly predicted as normal, no image was predicted as COVID-19, and seven and two images were misclassified as viral pneumonia and TB, respectively. For COVID-19, 294 images were predicted correctly; one image was incorrectly predicted as normal and another as viral pneumonia, and four images were incorrectly classified as Tb. For viral pneumonia, 283 images were predicted correctly, only 13 and 4 images were incorrectly predicted as normal and COVID-19, respectively, and no image was incorrectly classified as COVID-19. Similarly, 296 images were correctly predicted as Tb, and no images were incorrectly predicted as normal or viral pneumonia, but four images were misclassified as COVID-19. Table 9 shows the performance metrics for the best-performing model for the two classes (VGG19). Figure 5 shows the accuracy and loss curves of the VGG19 model for the two-class classifier over 50 epochs.

### 4.4. Five-Class Classifier

**Figure 6:**
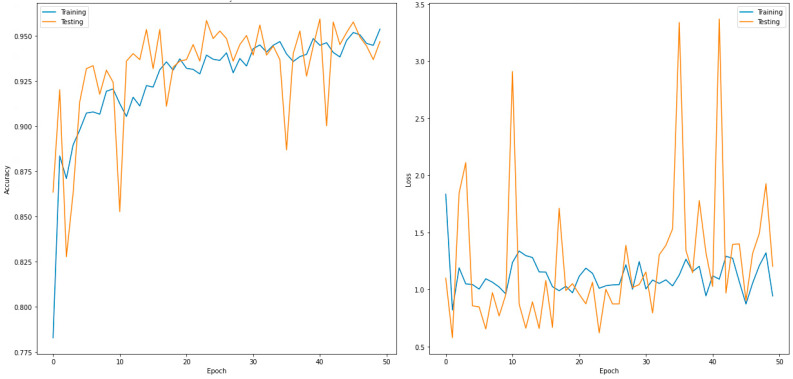
Accuracy (**left**) and loss curves (**right**) of DenseNet169 for five-class classifier.

This section describes the evaluated performance of the five-class classifier (normal, COVID-19, pneumothorax, bacterial pneumonia, and TB). Table 10 shows the validation confusion matrix for DenseNet201, which outperformed the other models. In total, 1200 images were used during the validation phase. These were further divided into 240 input images for normal, COVID-19, pneumothorax, bacterial pneumonia, and TB each. For the normal case, 234 images were correctly predicted as normal, no image was predicted as COVID-19 or pneumothorax, one image was misclassified as bacterial pneumonia, and five images were misclassified as Tb. For COVID-19, 230 images were correctly predicted as COVID-19, no images were predicted as either normal or bacterial pneumonia, and 10 images were misclassified as pneumothorax. For pneumothorax, 213 images were correctly predicted as pneumothorax, only one image was incorrectly predicted as bacterial pneumonia and Tb, no image was classified as normal, and 25 images were misclassified as COVID-19. Similarly, for bacterial pneumonia and Tb, 222 and 237 images, respectively, were correctly classified. Table 11 shows the performance metrics for the best-performing model for the two classes (DenseNet169). Figure 6 shows the accuracy and loss curves of the DenseNet169 model for the two-class classifier for 50 epochs.

### 4.5. Six-Class Classifier

**Figure 7:**
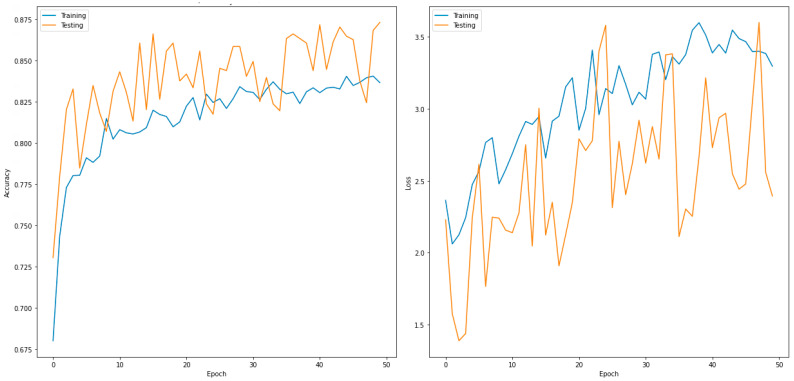
Accuracy (**left**) and loss curves (**right**) of DenseNet201 for six-class classifier.

In this section, we discuss the performance of the six-class classifier (normal, COVID-19, TB, bacterial pneumonia, viral pneumonia, and pneumothorax). Table 12 shows the validation confusion matrix for DenseNet201, which outperformed the other models. In total, 1440 images were used during the validation phase. These were further divided into 240 input images for each of the normal, COVID-19, TB, bacterial pneumonia, viral pneumonia, and pneumothorax cases. For the normal case, 227 images were correctly predicted as normal, one image was predicted as COVID-19 and another as bacterial pneumonia, three images were misclassified as Tb, and no images were classified as pneumothorax. For COVID-19, 212 images were predicted correctly, no images were predicted as normal or bacterial pneumonia, one image was misclassified as TB, two images were misclassified as viral pneumonia, and 25 images were misclassified as pneumothorax. Similarly, for Tb, bacterial pneumonia, viral pneumonia, and pneumothorax, 237, 170, 182, and 229 images were predicted correctly, respectively. Table 13 shows the performance metrics for the best-performing model for the two classes (DenseNet201). Figure 7 shows the accuracy and loss curves of the DenseNet201 model for the two-class classifier for 50 epochs.

Table 14 lists the classification performances of the proposed model in terms of the accuracy for the two-class, three-class, four-class, five-class, and six-class classifiers. For the two-class classifier, the accuracy of DenseNet169 and VGG19 was 99.83%, which is the same as that of the other models in the two-class classifier. For the three-class classifier, the accuracy of DenseNet201 was the highest among all models, and, for the four-class classifier, the accuracy of VGG19 was the highest. Similarly, for the five- and six-class classifiers, the accuracies of DenseNet201 were 94.66% and 87.29%, respectively, which are the highest among the models. Interestingly, for the two-class, three-class, and four-class classifiers, the accuracies were 99.83, 98.11%, and 97%, respectively. Similarly, for the five-class and six-class classifiers, the accuracies were 96.66% and 87.29%, respectively. The accuracy gradually decreased as the number of classes increased.

Table 15 compares the proposed model with existing schemes. For the two-class classifier, the accuracy of the model discussed by Chowdhury et al. [4] was 99.83%, and that by Ozturk et al. [20] was 98.08%, whereas the accuracy of the proposed model was 99.83%, which is 0.13% higher than that of the model proposed by Chowdhury et al. and 1.75% higher than that of the model proposed by Ozturk et al. For three classes, the accuracy of the model discussed by Chowdhury et al. [4] was 97.75%, that by Ozturk et al. [20] was 87.02%, and that by Khan et al. [24] was 95%; the accuracy of the proposed model was 98.11%, which is 0.16, 11.09, and 3.11% higher than these models, respectively. The proposed model evidently outperformed existing models.

## 5. Conclusions and Future Work

This study proposed a highly accurate TL-based system for the binary and multi-class classification of chest-related diseases in suspected patients. We created two balanced datasets, Dataset-1 and Dataset-2, and then classified chest-related diseases, including COVID-19, viral pneumonia, bacterial pneumonia, tuberculosis, pneumothorax, and pneumothorax, using nine pretrained models by dividing the diseases into five classes: two-class, three-class, four-class, five-class, and six-classifiers. For two, three, four, five, and six classes, our model achieved a maximum accuracy of 99.83, 98.11, 97.00, 94.66, and 87.29%, respectively. The proposed method outperformed existing state-of-the-art methods. In conclusion, this study investigated the use of artificial intelligence, specifically TL, in disease detection.

As an application, the proposed model can be used in challenging conditions where COVID-19, TB, and other chest-related diseases are major healthcare problems. The early detection of COVID-19 is necessary to reduce pressure on hospitals and medical facilities, as well as the burden on medical practitioners. Artificial intelligence and computer diagnosis play important roles in the early detection of diseases; therefore, this diagnostic tool may accelerate the screening time for COVID-19 and other chest-related diseases.

In the future, we plan to extend our study to other medical scenarios. In addition, we intend to train our model using extended datasets and perform binary and multi-class classification with different disease combinations that cover other chest-related diseases.

## Figures and Tables

**Table 1:** Comparison and characteristics of related studies.

Publications	Datasets	No of Classes	Train/Test Ratio	Models	Accuracy	Characteristics
Chowdhury et al. [4]	COVID-19 = 423 Viral pneumonia = 1485 Normal = 1579	2 __________ 3	80/20	MobileNetv_2, SqueezeNet, ResNet18, Inceptionv_3, ResNet101, Chex Net, VGG19, DenseNet201	99.7% ________ 97.95%	Unbalanced classes
Ozturk et al. [20]	COVID-19 = 125 Pneumonia = 500 Normal = 500	2 __________ 3	80/20	DarkCovidNet	98.08% ________ 87.02%	Unbalanced classes Low accuracy
Panwar et al. [23]	COVID-19 = 142 Normal 142	2	70/30	nCovNet	88%	Balanced classes with limited dataLow accuracy
Khan et al. [24]	COVID-19 = 284 Viral pneumonia = 327 Bacterial pneumonia = 330 Normal = 310	2 __________ 3 __________ 4	-	CoroNet	99% ________ 95% ________ 89.6%	Unbalanced classes Low accuracy
Wang et al. [26]	COVID-19 = 358 Pneumonia = 5538 Normal = 8066	3	-	COVID-net	93.3%	Unbalanced classes
Narin et al. [14]	COVID-19 = 341 Normal = 2800	2	80/20	ResNet101, ResNet_v2, Inception_v3, ResNet50	96.1%	Unbalanced classes
Ismael et al. [27]	COVID-19 = 180, Normal = 200	2	75/25	VGG16, ResNet50, ResNet18, ResNet101, VGG19	94.7%	Unbalanced classes Low accuracy

**Table 2:** Combined balanced datasets for three and five diseases: Dataset-1 and Dataset-2, respectively.

Disease	No. of Images for Dataset-1	No. of Images for Dataset-2
Normal	1500	1200
COVID-19	1500	1200
Viral pneumonia	1500	1200
Bacterial pneumonia	-	1200
Tuberculosis (Tb)	1500	1200
Pneumothorax	-	1200
Total number of images	6000	7200

**Table 3:** Assignment of diseases according to different classifiers.

Classes	Diseases
Two-class classifier	Normal and COVID-19
Three-class classifier	Normal, COVID-19 and viral pneumonia
Four-class classifier	Normal, COVID-19, viral pneumonia and Tb
Five-class classifier	Normal, COVID-19, bacterial pneumonia, Tb, and pneumothorax
Six-class classifier	Normal, COVID-19, bacterial pneumonia, viral pneumonia, Tb and pneumothorax

**Table 4:** Validation confusion matrix of DensNet169 for two-class classifier.

**True Label**		**Predicted Label**	
	**(No. of Images = 600)**	**Normal**	**COVID-19**
	Normal	300	0
	COVID-19	1	299

**Table 5:** Performance metrics of DenseNet169 for two-class classifier.

Performance Metrics	Achieved Score (%)
Accuracy	99.833
F2-Score	99.831
Precision	99.833
Recall	99.831
Specificity	99.834

**Table 6:** Validation confusion matrix of DenseNet201 for three-class classifier.

**True Label**	**Predicted Label**			
	**(No. of Images = 900)**	**Normal**	**COVID-19**	**Viral Pneumonia**
	Normal	291	0	9
	COVID-19	1	297	2
	Viral pneumonia	5	0	295

**Table 7:** Performance metrics of DenseNet201 for three-class classifier.

Performance Matrices	Achieved Score (%)
Accuracy	98.111
F2-Score	98.114
Precision	98.128
Recall	98.111
Specificity	99.055

**Table 8:** Validation confusion matrix of VGG19 for four-class classifier.

**True Label**	**Predicted Label**				
	**(No. of Images = 1200)**	**Normal**	**COVID-19**	**Viral Pneumonia**	**Tb**
	Normal	291	0	7	2
	COVID-19	1	294	1	4
	Viral pneumonia	13	4	283	0
	Tb	0	4	0	296

**Table 9:** Performance metrics of VGG19 for four-class classifier.

Performance Metrics	Achieved Score (%)
Accuracy	97.000
F2-Score	97.001
Precision	97.006
Recall	97.000
Specificity	99.000

**Table 10:** Validation confusion matrix of DenseNet169 for five-class classifier.

**True Label**	**Predicted Label**					
	**(No. of Images = 1200)**	**Normal**	**COVID-19**	**Pneumothorax**	**Bacterial Pneumonia**	**Tb**
	Normal	234	0	0	1	5
	COVID-19	0	230	10	0	0
	Pneumothorax	0	25	213	1	1
	Bacterial pneumonia	13	3	0	222	2
	Tb	2	1	0	0	237

**Table 11:** Performance metrics of DenseNet169 for five-class classifier.

Performance Metrics	Achieved Score (%)
Accuracy	94.666
F2-Score	94.698
Precision	94.827
Recall	94.666
Specificity	98.666

**Table 12:** Validation confusion matrix of DenseNet201 for six-class classifier.

**True Label**	**Predicted Label**						
	**No of Images = 1440**	**Normal**	**COVID-19**	**Tb**	**Bacterial Pneumonia**	**Viral Pneumonia**	**Pneumothorax**
	Normal	227	1	3	1	8	0
	COVID-19	0	212	1	0	2	25
	Tb	0	1	237	0	0	2
	Bacterial pneumonia	6	0	0	170	62	2
	Viral pneumonia	5	2	0	51	182	0
	Pneumothorax	0	8	3	0	0	229

**Table 13:** Performance metrics of DenseNet201 for six-class classifier.

Performance Metrics	Achieved Score (%)
Accuracy	87.291
F2-Score	87.304
Precision	87.356
Recall	87.291
Specificity	97.458

**Table 14:** Accuracy (%) of the proposed model for different classes using different deep learning networks.

Models	Two-Class Classifier	Three-Class Classifier	Four-Class Classifier	Five-Class Classifier	Six-Class Classifier
DenseNet201	99.50	98.11	96.75	94.66	87.29
DenseNet169	99.83	97.33	96.75	94.25	84.16
DenseNet121	99.50	96.88	96.00	91.41	85.34
VGG16	99.83	97.77	95.49	93.58	84.79
VGG19	99.83	97.66	97.00	94.41	84.72
ResNet50	97.33	85	82.16	66.50	64.72
ResNet101	90.66	86.44	79.66	75.33	65.83
NasNet-Mobile	99.33	96	95.33	93.33	84.30
MobileNet_v2	99.66	97.77	96.75	93.66	81.38

**Table 15:** Comparison of proposed approach with the existing methods.

Publications	No of Classes	Dataset	Accuracy
Chowdhury et al. [4]	2	Unbalanced	99.7%
	_____________		_________
	3		97.95%
Ozturk et al. [20]	2	Unbalanced	98.08%
	_____________		_________
	3		87.02%
Panwar et al. [23]	2	2	88%
Khan et al. [24]	2	Unbalanced	99%
	_____________		_________
	3		95%
	_____________		_________
	4		89.6%
Wang et al. [26]	2	Unbalanced	93.3%
Narin et al. [14]	2	Unbalanced	96.1%
Ismael et al. [27]	2	Unbalanced	94.7%
Proposed work (DenseNet169)	2	Balanced	99.83%
Proposed work (DenseNet201)	3	Balanced	98.11%
Proposed work (VGG19)	4	Balanced	97%
Proposed work (DenseNet169)	5	Balanced	94.66%
Proposed work (DenseNet201)	6	Balanced	87.29%
